# A Generalized Crystallization Protocol for Scalable Deposition of High‐Quality Perovskite Thin Films for Photovoltaic Applications

**DOI:** 10.1002/advs.201901067

**Published:** 2019-06-25

**Authors:** Fei Guo, Shudi Qiu, Jinlong Hu, Huahua Wang, Boyuan Cai, Jianjun Li, Xiaocong Yuan, Xianhu Liu, Karen Forberich, Christoph J. Brabec, Yaohua Mai

**Affiliations:** ^1^ Institute of New Energy Technology College of Information Science and Technology Jinan University Guangzhou 510632 China; ^2^ Nanophotonics Research Center Shenzhen Key Laboratory of Micro‐scale Optical Information Technology Shenzhen University Shenzhen 518060 China; ^3^ National Engineering Research Center for Advanced Polymer Processing Technology Zhengzhou University Zhengzhou 450002 China; ^4^ Institute of Materials for Electronics and Energy Technology (i‐MEET) Friedrich‐Alexander University Erlangen‐Nürnberg Martensstrasse 7 91058 Erlangen Germany; ^5^ Helmholtz Institute Erlangen‐Nürnberg for Renewable Energy (IEK‐11) Forschungszentrum Jülich GmbH Immerwahrstraße 2 91058 Erlangen Germany

**Keywords:** blade coating, one‐step, perovskites

## Abstract

Metal halide perovskite solar cells (PSCs) have raised considerable scientific interest due to their high cost‐efficiency potential for photovoltaic solar energy conversion. As PSCs already are meeting the efficiency requirements for renewable power generation, more attention is given to further technological barriers as environmental stability and reliability. However, the most major obstacle limiting commercialization of PSCs is the lack of a reliable and scalable process for thin film production. Here, a generic crystallization strategy that allows the controlled growth of highly qualitative perovskite films via a one‐step blade coating is reported. Through rational ink formulation in combination with a facile vacuum‐assisted precrystallization strategy, it is possible to produce dense and uniform perovskite films with high crystallinity on large areas. The universal application of the method is demonstrated at the hand of three typical perovskite compositions with different band gaps. P‐i‐n perovskite solar cells show fill factors up to 80%, underpinning the statement of the importance of controlling crystallization dynamics. The methodology provides important progress toward the realization of cost‐effective large‐area perovskite solar cells for practical applications.

## 1 Introduction

Deposition of photoactive absorbers by scalable printing methods is an attractive approach to realize the cost potential of emerging photovoltaic technologies toward industrial applications.^1^, ^2^ As state‐of‐the‐art direct band gap semiconductors, metal halide perovskites that can be simply fabricated from solution are currently in the focus of the photovoltaic community.^3^, ^4^ The superior optoelectronic merits of high absorption coefficient, large ambipolar mobility, and long charge carrier lifetime resulted in power conversion efficiencies (PCE) of perovskite solar cells rocketing from 3.8% to the level of 23.7% within a decade.^5^ Despite these impressive achievements, most of the reported high efficiency devices were fabricated by spin‐coating which allows to prepare small‐size perovskite films using small volume of solution, although most solution is wasted during spinning.^6^, ^7^ Nevertheless, the biggest bottleneck of spin‐coating is that it is neither scalable nor can be translated to other scalable deposition processes. To further advance the technology toward industrial aspirations, it is highly imperative to redevelop facile and robust deposition protocols that can be used for fabrication of perovskite films via scalable printing methods.

Recently, several scalable deposition techniques have been developed to prepare perovskite films, such as spray‐coating,^8^ doctor‐blading,^9^, ^10^, ^11^, ^12^, ^13^, ^14^ slot‐die coating,^2^, ^15^, ^16^, ^17^ and so on. Among these printing methods, doctor blading is one of the most promising lab techniques because it is easy to handle with minimized material waste and, importantly, it can be readily transferred to slot‐die coating for sheet‐to‐sheet or roll‐to‐roll volume manufacturing. Blade coating can be carried out at elevated substrate temperatures which has been maturely adopted to prepare organic optoelectronic devices,^18^, ^19^ but, is limited in controlling the ink temperature. Recently, several groups have reported progress in blade coating highly qualitative perovskite films at substrate temperatures over 100 °C.^9^, ^10^, ^11^, ^12^, ^20^, ^21^, ^22^ However, it has been noticed that elevated processing temperatures close to or even higher than the temperature required for perovskite crystallization can lead to simultaneous solvent evaporation and crystal growth which, in turn, would constrain the processing window as well as the repeatability of device fabrication. In this context, development of a controlled crystallization protocol that is compatible with low‐temperature deposition of precursor films is highly demanded for large‐scale perovskite thin film manufacture.

Crystallization is a complicated process which is aimed at creating a crystalline material from liquid, gaseous, or amorphous solid systems. Nucleation and crystal growth are the two fundamental steps of vital importance which usually take place during the formation of a supersaturated state. Specific for polycrystalline perovskite thin‐film growth, it generally involves three steps: i) deposition of a liquid precursor film onto a substrate, ii) drying of the wet film to reach a supersaturation followed by nucleation, and, iii) thermal annealing, where necessary, to facilitate perovskite crystal growth. The paramount challenge to realize high‐quality perovskite films via printing methods is that the two critical steps, nucleation and crystal growth, usually take place in seconds and often simultaneously, making perovskite crystallization intractable. In the classical antisolvent crystallization strategy for spin‐coated perovskite films, it is commonly recognized that a temporal intermediate phase is created upon applying an immiscible solvent at a critical time of high‐speed substrate rotation.^6^ This frozen intermediate stage alleviates the otherwise fast nucleation and crystal growth, leading to uniform and dense perovskite films upon a thermal annealing. Despite the ground success of spin‐coating for processing perovskite films in the lab, the well‐developed antisolvent strategy is extremely complex to transfer to large area printing lines due to the excess and spilling of washing solvents.

In this paper, we report a generic crystallization strategy that allows depositing high‐quality perovskite thin films by blade coating at ambient temperature. We highlight that the key to the successful implementation of the technology relies on a combination of judiciously engineered precursor formulations and a deliberately designed intermediate crystallization step. Specifically, incorporation of a small amount of additive methylammonium chloride (MACl) into the perovskite precursor not only allows for fine tuning the morphology and crystallinity of the film but, more importantly, the presence of MACl can effectively suppress the formation of nonperovskite δ‐phase in the formamidinium and cesium (FACs)‐involved material system. Another central design of our protocol is the deployment of a vacuum‐based precrystallization procedure, which is decisive to create intermediate phases by removing the excess solvent of the freshly printed films. The resulting intermediate stage guarantees that the precursor deposition is kept separated from the subsequent thermal‐annealing based crystallization, leading to the growth of high‐quality perovskite films in a controlled manner. To demonstrate the universal application of the protocol, we prepared three perovskite material systems with exemplary compositional cations and halides including: MAPbI_3_ (1.58 eV), FA_0.95_Cs_0.05_PbI_3_ (1.52 eV), and FA_0.6_MA_0.4_Pb(I_0.6_Br_0.4_)_3_ (1.76 eV). All three compositions are demonstrated with blade coating to reach a device performance on par with spin coating. In particular, a high fill factor (FF) up to 80% (obtained in 0.09 cm^2^ device) and large‐area module (4 × 4 cm^2^) with negligible potential losses demonstrated the high quality of the perovskite films and the reliability of the crystallization protocol.

## 2 Results and Discussions

**Figure 1:**
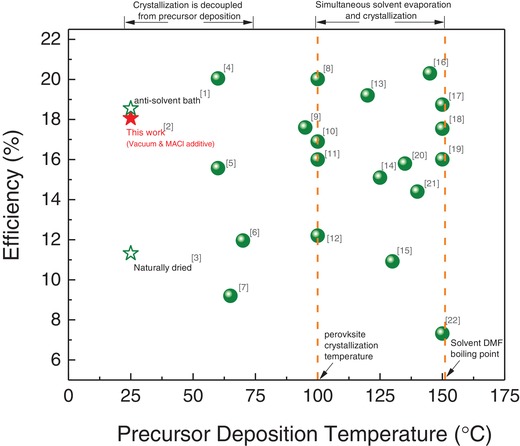
The efficiency versus precursor deposition temperature for the preparation of perovskite solar cells via scalable blade coating or slot‐die coating. It is noted that, although the work [1] (in the Supporting Information) reported the room‐temperature deposition of perovskite precursor, the crystallization was carried out in an antisolvent bath which is not ideal for practical manufacture.^13^

To underline the significance of this work, we first compare the “efficiency” versus “precursor deposition temperature” for the scalable fabrication of perovskite solar cells from previous reports. The collected data points are plotted in **Figure** **1** with the specific values and the corresponding references summarized in Table S1 (Supporting Information). From the plot, we can see that most of the perovskite solar cells were deposited at temperatures over 100 °C. As mentioned above, perovskite crystallization can take place concurrently with the solvent evaporation during precursor deposition at such high temperatures, which makes it challenging to control the crystal morphology. This deficiency will be addressed in this work by decoupling the precursor deposition with the subsequent thermal annealing via additive‐ and vacuum‐assisted crystallization strategy. Our technology ultimately provides a simple yet reliable crystallization protocol for blade deposition of perovskite solar cells with a wide range of material composites.

### 2.1 Precursor Ink Deployment and Film Characterizations

We start with a rational design of perovskite precursor ink by means of additive engineering. Previous works have shown that chemical additives such as organic molecules,^23^, ^24^, ^25^, ^26^ inorganic salts,^27^ and ionic liquids^28^ etc. incorporated into perovskite precursors can have significant influence on the crystal quality and overall morphology of spin‐coated perovskite films. Depending on the functionality of additives, uniform perovskite films can be obtained by providing homogeneous nucleation sites, and prolonged crystallization time can result in increased crystal size.^23^, ^24^, ^27^ Inspired by these reports, we anticipate that the crystallization dynamics of perovskite films deposited by scalable methods can analogously be modulated by incorporating small amounts of additives into the precursor. This is the primary motivation that inspires us to formulate precursor inks by additive engineering. We choose MACl as the additive for our perovskite systems primarily based on the following reasons. First, MACl can effectively slow down the perovskite crystallization rate, which is expected to provide a viable approach to manipulate film morphology.^29^ Second, the volatility of MACl allows the excessive additives to be evaporated from the perovskite film during thermal annealing.^30^ Consequently, the phase purity and device performance will not be impaired by additives in the perovskite film. Significantly, we found that the incorporation of MACl plays an essential role in suppressing the formation of the yellow δ‐phase during processing FACs‐involved perovskite films.

To begin with, we employ MAPbI_3_ as our model material system in consideration of its simpler chemical composition compared to the multiple‐cation and mixed‐halide perovskites. It is thus expected to offer a facile platform in understanding the thin‐film formation mechanism of the additive‐ and vacuum‐assisted crystallization. We incorporated different levels of MACl, from 10% to 100% mole ratio relative to MAPbI_3_, into the precursor solution and deposited the precursor films by doctor‐blade coating. The eventual crystalline perovskite films were obtained by vacuum extraction of the freshly deposited precursor films followed by a thermal‐annealing step. Detailed procedures for perovskite film fabrication are presented in the Experimental Section and will be discussed in the next section.

**Figure 2:**
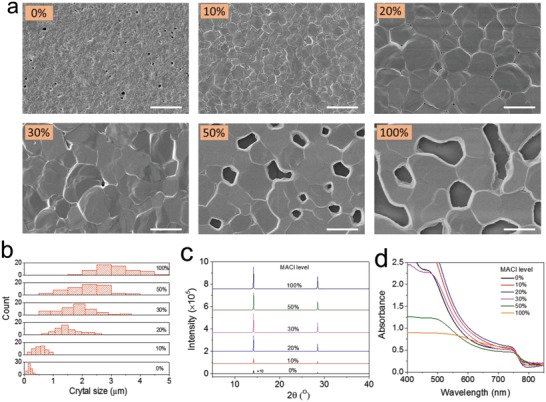
a) Top‐view SEM images of perovskite films with different amounts of MACl prepared by blade coating; scale bars are 2 µm. b) Statistical distribution of the grain sizes of the perovskite films with different amounts of MACl additives. A total of 60 crystal grains are counted for each statistic plot. c) XRD patterns and d) absorption spectra of blade‐coated perovskite films with different amount of MACl.

We evaluate the robustness of our crystallization protocol by scrutinizing the microstructure, crystallinity, and optical properties of the polycrystalline MAPbI_3_ films processed by blade coating. **Figure** **2**a shows the top‐view scanning electron microscopy (SEM) images of the perovskite films fabricated from precursor inks with different amounts of MACl. It is obvious that the crystal grain size enlarges substantially from ≈200 nm to over 4 µm with increasing content of MACl (Figure 2b), which confirms that the MACl additive plays an important role in nucleation and crystal growth. It has been demonstrated that perovskite ingredients dissolved in solution are mainly in the form of aggregated colloid particles rather than movable ions.^31^ We therefore correlated the crystal size evolution to the coordination between the precursor solute and solvent, as manifested by the observation of Tyndall effect in the precursor solutions (Figure S1, Supporting Information). It is also apparent that the additive has a significant effect on surface coverage of the films. Without MACl incorporation, several small holes with diameters of ≈200 nm are randomly distributed across the film. The existence of holes can deteriorate device performance by forming shunts. Remarkably, the incorporation of a small amount of 10% MACl produces uniform and dense perovskite films with complete coverage. Further increasing the MACl content to larger crystals but also introduces big uncovered areas, particularly for MACl loads of more than 50%. The formation of such voids presumedly results from the release of excess MACl during annealing processes. Cross‐sectional SEM images shown in Figure S2 of the Supporting Information evidence vertically aligned monolayer crystals with grain boundaries formed throughout the cross section for all levels of MACl content. In contrast, the perovskite film processed without additive shows randomly stacked small‐size grains. We note that enlarged crystal grains with vertically stacked boundaries could benefit charge transport with minimal recombination at grain boundaries as the photogenerated carriers can travel through the active layer and reach the corresponding charge carrier extraction interfaces.

The structural and optical properties of the prepared perovskite films were inspected by X‐ray diffraction (XRD) and ultraviolet‐visible (UV–vis) absorption analyses with results presented in Figure 2c,d, respectively. We fixed the thickness of perovskite films at ≈450 nm by keeping all the deposition parameters constant. From the XRD patterns, we can see that the diffraction peaks at 14.1° and 28.2°, respectively, indexing to the (110) and (220) planes of MAPbI_3_ are present for all perovskite films. The two diffraction peaks become much sharper and more intense with higher loads of additives, which is consistent with the enlarged grain sizes. For a better comparison, the XRD spectra were plotted with Y‐axis in logarithm scale (Figure S3, Supporting Information), where we can see that four additional diffraction peaks at 20.05° (112), 23.51° (211), 24.5° (202), and 31.92° (312) were observed for the perovskite without MACl additive. Upon addition of MACl, the intensity of the peaks slightly reduced in 10% MACl processed film which ultimately disappeared in the films with MACl loads higher than 20%. Particularly, the presence of the single two peaks at 2θ = 14.1° and 28.4° for the films with higher levels of MACl indicates a preferential crystal growth of (110) faces parallel to the substrate. Zoom‐in spectra of the (110) and (220) planes shown in Figure S4 of the Supporting Information evidences negligible shift of the peaks, underlining that the addition of MACl does not significantly impact the MAPbI_3_ crystal structure. Figure S5 of the Supporting Information displays the full width at half maximum (FWHM) of the (110) and (220) peaks of MAPbI_3_ films as a function of MACl addition content, suggesting that MACl loads from 10% to 30% yield films with optimum crystallinity. From the UV–vis spectra shown in Figure 2d, we can see that the absorption of the perovskite films prepared with 10% to 30% MACl additives is slightly stronger than the pristine film. When MACl contents increased to 50% and higher, the absorption below 600 nm is strikingly reduced primarily due to the existence of large voids.

Considering that MACl has been previously reported to modulate the crystal morphology of the perovskite films based on spin‐coating process, the direct transition of the precursor ink from spin‐coating to our scalable printing methods demonstrated the unique advantages of our scalable deposition technology. This is indeed of vital importance for industrial production given that precursor inks developed for lab‐scale spin‐coating process can be directly transferred to scalable printing lines without redesigning the formulations. To further illustrate the generic application of the additive engineering in modulation of the crystal morphology of the printed perovskite films, we further prepared perovskite films with incorporation of additives of ammonium thiocyanate (NH_4_SCN) and potassium thiocyanate (KSCN) by blade‐coating.^32^ As presented in Figure S6 of the Supporting Information, the SEM images evidence that the addition of small amounts of NH_4_SCN and KSCN into the precursor solution can have analogous impacts on the film morphologies of blade‐coated perovskite thin films.

### 2.2 Controlled Perovskite Crystallization

**Figure 3:**
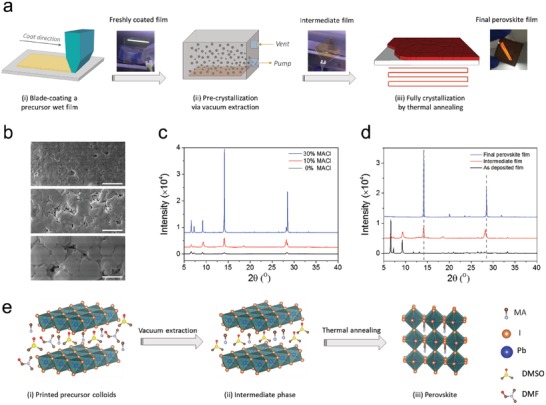
a) Schematic illustration of the one‐step deposition of a perovskite film via blade coating, where a vacuum extraction step ii) is used to create an intermediate film by removing excess solvent of the freshly printed wet film i), and fully crystalline perovskite film was obtained by a subsequent thermal annealing procedure iii). Digital photographs in (a) are, from left to right, the as‐deposited precursor wet film, intermediate film, and final crystalline perovskite film all on glass substrate. b) SEM images of intermediate MAPbI_3_ films processed without (top), with 10% (middle), and 30% (bottom) MACl additives; Scale bars are 1 µm. c) XRD spectra of the corresponding intermediate films in (b). d) XRD spectra of the as‐deposited MAPbI_3_ (10% MACl) precursor film, the intermediate film, and the final crystallized film subjected to an annealing step at 100 °C for 10 min. e) Scheme of the MAPbI_3_ perovskite thin film forming process. i) PbI_2_, MAI, and MACl in the mixture solvent of DMF and DMSO; ii) intermediate phase formation after vacuum extraction; iii) MAPbI_3_ perovskite thin film forms after thermal annealing.

We now turn to illustrate the intractable yet of uttermost importance of the crystallization mechanisms for perovskite thin films prepared by blade coating. **Figure** **3**a illustrates the schematic procedure for the fabrication of perovskite thin films by blade deposition and the proposed vacuum‐assisted crystallization protocol. The first step involves the deposition of a perovskite precursor film on the substrate via doctor‐blade coating. The freshly deposited precursor film contains a large amount of solvent which requires to be judiciously removed as it would lead to unpredicted crystallization due to the simultaneous evaporation of solvent and solute upon thermal annealing. As mentioned earlier, this process is distinctly different to the previously reported approach to blade‐coating at elevated temperatures, which are close to or exceeding the boiling point of solvents.^9^, ^10^, ^11^, ^12^, ^20^, ^21^, ^22^ The crystallization process driven by such high temperatures will take place concurrently during precursor deposition. High temperature blading has the inherent disadvantage that film deposition is not decoupled from film drying and crystallization. With nucleation, precipitation, and mass transport happening simultaneously, process control becomes challenging and may result in undesired reproducibility or yield.

The classical antisolvent strategy provides a sound hint illustrating the importance of slowing down perovskite crystallization dynamics, which is realized by creating an intermediate phase upon pouring an immiscible solvent at a delayed spinning stage.^6^ Enlightened by the antisolvent approach, we sought to achieve such an intermediate stage via vacuum extraction to remove excess solvent of the freshly deposited wet films. The resulting intermediate film could effectively separate the precursor film deposition from subsequent thermal annealing (Figure 3a), thus, allowing the precursor deposition to be carried out at room temperature. Finally, highly crystallized perovskite films with shiny surface were obtained by annealing the intermediate films at 100 °C for 10 min. We note that although vacuum extraction has been reported by several groups, it has been predominantly explored for preparation of perovskite films based on spin‐coating.^25^, ^33^, ^34^, ^35^ In combination with additive engineering, in the present work, we endeavor to exploit a generalized method that allows to precisely manipulate the morphology and crystallinity of perovskite films for a wide range of material compositions.

We highlight that the deployment of vacuum extraction and the incorporation of MACl are the two paramount important factors affecting the quality of the eventual perovskite films. As indicated in Figure S7 of the Supporting Information, thermally induced crystallization from the naturally dried perovskite precursors (without the vacuum extraction step) yield coarse surfaces with randomly aligned textured structure, regardless of MACl incorporation. In contrast, prior to thermal annealing and after a moderate vacuum process at 1000 Pa for 2 min, the resulting perovskite films with different dimensions (as large as 38.5 cm^2^) show a mirror‐like smooth surface and clear nonscattered transmitted light (Figure S8, Supporting Information). Significantly, we observed an obvious color transformation from transparent yellow of the wet precursor film to shiny orange of the intermediate film during the vacuum process at around 1 min (Figure 3a). This observation was quite similar to the spin‐coated perovskite films based on the antisolvent strategy.^6^ Moreover, we found that the higher the level of MACl additive, the browner the intermediate films and the shorter time required for color change. We speculate that a certain degree of crystallization of perovskite took place in the intermediate films which was driven by the formation of supersaturation due to the vacuum removal of the excess solvent. To verify this hypothesis, we conducted SEM and XRD analyses to unveil the morphology and phase evolution of the intermediate films processed with 0%, 10%, and 30% MACl additives. SEM images shown in Figure 3b evidence that the intermediate film without additive is composed mainly of amorphous regions along with randomly aligned textured crystals. With incorporation of 10% MACl, noticeable cluster of grains appeared which are located on top of the textured crystals. Upon further increasing the MACl content to 30% cluster of grains become larger. The enlarged crystal gains in the intermediate films match well with the grain size evolution of the final crystalline perovskites. The formation of the perovskites was further confirmed by XRD measurement which is shown in Figure 3c. One can see that the characteristic perovskite diffraction peaks at 14.1° and 28.4° are present for all the three films and that the peaks become more intense and narrower with higher MACl content. These results attest that a mild vacuum at room temperature can induce the perovskite formation independent of additive incorporation, while the presence of MACl can promote the transformation of the precursor aggregates to crystalline perovskite possibly by reducing the activation energy for nucleation and crystallization.

In view of the importance of intermediate phases in regulating the morphology and crystallinity of the eventual perovskite films, we now take a close look at the structural evolution of the precursor films at different processing stages. We note that dimethylformamide (DMF):dimethyl sulfoxide (DMSO) with volume ratio of 4:1 was used as a solvent system for preparing our perovskite precursor inks, and it has been previously demonstrated that strong coordination between DMSO and PbI_2_ via intercalation can benefit the perovskite film growth by retarding the reaction between the PbI_2_ and MAI.^6^, ^36^ As indicated in Figure 3d, the XRD spectrum of the naturally dried precursor film (without vacuum extraction step) shows three diffraction peaks at low angles of 6.63°, 7.25°, and 9.23°. This crystalline complex can be ascribed to the intercalation of MAI and DMSO into layered PbI_2_ with a molecular structure of MA_2_Pb_3_I_8_·2DMSO, which has been resolved by several groups.^6^, ^37^, ^38^, ^39^ Interestingly, upon a vacuum extraction process, these small angle peaks were obviously suppressed, which can be attributed to the collapse of some complex due to removal of DMSO. Simultaneously, two perovskite characteristic peaks (14.1° and 28.4°) emerged. It is also surprising to perceive that crystalline PbI_2_ (diffraction 2θ = 12.7°) was negligible in both the intermediate film and the naturally dried precursor film. This observation implies that the PbI_2_ in the intermediate film predominately forms crystalline MA_2_Pb_3_I_8_·2DMSO complexes rather than bulk PbI_2_ crystals. We further confirmed the presence of DMSO and MACl in the intermediate film with Fourier transform infrared spectroscopy (FTIR) and X‐ray photoelectron spectroscopy (XPS) (Figure S9, Supporting Information). When the intermediate film was subjected to an annealing step, highly crystalline perovskite was obtained, as evidenced by the disappearance of the low angle peaks with emergence of the two intense and sharp perovskite peaks (14.1° and 28.4°).

Having revealed the structural and morphological evolution of the printed films at different stages, the main reaction processes involved in our crystallization protocol can be proposed as follows (which is schematically illustrated in Figure 3e). i) The perovskite precursor film deposited by blade‐coating contains excess solvent and the precursor ingredients in the wet film exist mainly in the form of aggregated colloid particles. ii) Once the wet precursor film is subjected to a vacuum extraction, the solvent DMF and excess DMSO are extracted from the wet film. The vacuum process creates a supersaturation stage, which not only transforms the precursor colloids to the intermediate film (MA_2_Pb_3_I_8_·2DMSO), but simultaneously induces the formation of crystalline perovskite. iii) At last, applying a thermal annealing to the intermediate film, the MACl volatilized from the system and the residual DMSO was liberated from the MA_2_Pb_3_I_8_·2DMSO complex and, simultaneously, PbI_2_ reacted with MAI to produce tetragonal perovskite films.

### 2.3 Photovoltaic Performance at Different Device Scales

**Figure 4:**
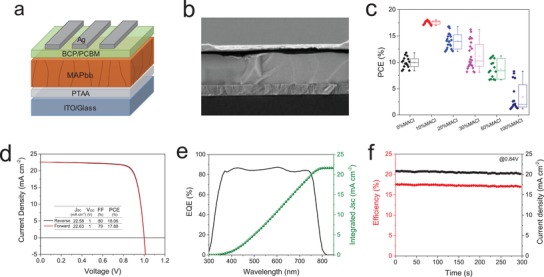
a) Schematic diagram of the planar p‐i‐n structure perovskite solar cells. b) Cross‐sectional SEM image of a blade‐deposited MAPbI_3_ (10% MACl) perovskite solar cell, scale bar is 500 nm. c) Statistic PCE of the blade‐coated MAPbI_3_ solar cells with different levels of MACl content. d) *J–V* characteristic of the best MAPbI_3_ (10% MACl) device scanned from both forward and reverse scans. e) EQE spectrum of a MAPbI_3_ (10% MACl) solar cell. f) The stabilized power output of a typical MAPbI_3_ cell monitored over time near the maximum power point.

To evaluate the performance of the doctor‐blade deposited MAPbI_3_ films for photovoltaic applications, we fabricated solar cells with a device architecture of glass/ITO/PTAA/MAPbI_3_/PCBM/BCP/Ag (**Figure** **4**a). The cross‐sectional SEM image of a complete device with 10% MACl additive is shown in Figure 4b, which evidences micrometer‐scale perovskite crystals monolithically aligned throughout the device. Figure 4c and Figure S10 of the Supporting Information present the statistic photovoltaic performance of the prepared MAPbI_3_ solar cells with different amounts of MACl. Solar cells without MACl incorporation show rather low efficiencies of around 10%. Remarkably, 10% MACl mediated perovskites deliver the highest overall performance of around 17.5%, particularly in terms of high *V*_OC_ of ≈1.00 V and fill factors (FF) up to 80%. However, when the MACl content exceed 20%, all the photovoltaic parameters tend to decline, which can be attributed to the presence of shunts and low light harvesting capacities due to the existence of large voids in the perovskites. Figure 4d shows the current density–voltage (*J*–*V*) curves of the best solar cell processed from 10% MACl, which gives an efficiency of 18.06% with *J*_SC_ of 22.58 mA cm^−2^, *V*_OC_ of 1.00 V and FF of 80% obtained from the reverse scan. It is also noticeable that the 10% MACl mediated solar cells show no hysteresis which is in striking contrast with the solar cells processed without and with MACl contents higher than 20% (Figure S11, Supporting Information). The high performance along with free of hysteresis of the device prepared from 10% MACl additive can be attributed to the high crystallinity and absence of pin‐hole related recombination paths.^40^ This can be evidenced from the SEM images (Figure 1a) that 10% MACl processed perovskite film showed complete coverage with compact crystal grains, while lots of pinholes appeared in the sample without MACl incorporation and the pinholes become larger with higher MACl additives. In Figure 4e, we show the external quantum efficiency (EQE) spectrum yielding an integrated *J*_SC_ value of 21.85 mA cm^−2^, which is in good agreement with the *J–V* measurement, with a discrepancy below 5%. We tested the steady state power output at maximum power point (MPP) of a blade‐deposited solar cell and the result shows a small decline (current‐density decreased from 20.7 to 20.2 mA cm^−2^) under continuous tracing under 1 sun illumination for 300 s (Figure 4f). To get a more comprehensive view on the stability of our solar cells, we performed a prolonged operational stability test of a device over a period of more than an hour.^41^, ^42^, ^43^ As indicated in Figure S12 of the Supporting Information, the solar cell underwent a 6.3% degradation after 66 min continuous illumination, suggesting acceptable stability of our blade‐deposited perovskite solar cells. Furthermore, the shelf stability of the devices stored in N_2_‐filled glovebox was also evaluated. The result shown in Figure S13 of the Supporting Information indicates excellent shelf stability of the blade‐deposited perovskite solar cells as almost no performance losses were observed over a month's storage.

**Figure 5:**
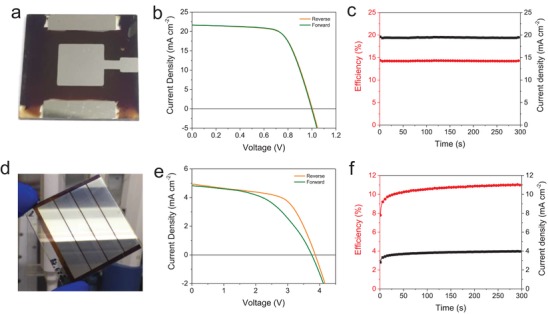
a) Digital photograph, b) *J–V* curves, and c) stabilized current‐density and PCE output of a typical blade‐coated MAPbI_3_ solar cell (10% MACl additive) with active area of 1 cm^2^. d) Digital photograph, e) *J–V* curves, and f) stabilized current‐density and PCE output of the best blade‐coated MAPbI_3_ solar module (10% MACl additive) with active area of 10.08 cm^2^ and four cells connected in series.

To demonstrate the uniformity of the blade‐coated perovskite films, we continue to produce solar cells with an active‐area of 1 cm^2^ (**Figure** **5**a). Figure 5b shows the measured *J–V* curves which gives a PCE of 14.72% with *V*_OC_ of 1.01 V, *J*_SC_ of 21.70 mA cm^−2^, FF of 67.15% (forward scan), and again the large‐area device showed negligible hysteresis. The stabilized photocurrent of the device at the MPP (biased at 0.74 V) are shown in Figure 5c, yielding a stabilized PCE of 14.3%. The statistic device performance based on 15 solar cells from two batches is shown in Figure S13 of the Supporting Information, suggesting excellent reproducibility of the device fabrication and high quality of the blade‐coated perovskite films. Compared with the small‐size devices, the performance losses mainly come from the lower FF.^44^ We therefore derived the series resistance (*R*_S_) and parallel resistance (*R*_P_) of the 1 cm^2^ device from the measured *J–V* curves. As displayed in Figure S14 and Table S2 of the Supporting Information, the calculated *R*_S_ is as high as 8.25 Ω cm^2^, which is more than four times larger than the small‐size device, whereas the *R*_P_ values for both devices are more than 1000 Ω cm^2^. These results indicate that, other than current leakage through shunts, the large series resistance mainly contributed to the lower FF of the 1 cm^2^‐size solar cells, which was probably resulted from resistance losses due to the high sheet resistance of ITO electrode (17 Ω sq^−1^). To confirm this hypothesis, we have built another 1 cm^2^ solar cell on PTAA‐coated ITO electrodes with a lower sheet resistance of 7 Ω sq^−1^. As can be seen in Figure S14 of the Supporting Information, the device deposited on 7 Ω sq^−1^ ITO electrode shows a lower series resistance than that on 17 Ω sq^−1^ ITO. Accordingly, an enhanced FF of 72.6% was obtained (Table S2, Supporting Information).

Manufacturing of large‐area modules is actively pursued on the way of realizing practical applications of thin‐film photovoltaic technologies. The general challenge, however, is the quality and uniformity of the absorber films on large‐scale levels. The highly reproducible fabrication of 1 cm^2^ cells by blade‐coating further encouraged us to manufacture modules with larger areas. We designed and fabricated 4 × 4 cm^2^ modules with four cells monolithically interconnected in series (Figure 5d). The module has an active area of 10.08 cm^2^ and an interconnection area of 2.88 cm^2^, leading to geometric fill factor of 77.78% (Figure S15, Supporting Information). The *J–V* curves of the module in Figure 5e give a PCE of 11.25% (*J*_SC_ = 4.93 mA cm^−2^, *V*_OC_ = 3.87 V, FF = 59%) from the reverse scan. However, a noticeable hysteresis was observed which is probably due to the inferior interconnection formation. We also tracked the PCE of the module near the MPP (biased at 2.85 V). The result indicates a slight current density increase during the illumination, leading to an efficiency of 11.01% under continuous light‐soaking for 300 s (Figure 5f). It is worth noting that the voltage of the four‐cell module reaches 3.87 V, suggesting negligible *V*_OC_ losses (an average of 32.5 mV loss for the individual sub‐cell) which again validates the high quality of the blade‐coated perovskite film over the large area. The relatively low FF of the module may originate from the high resistance for charge recombination in the interconnection area where the P2 line was scribed manually with a tweezer.

### 2.4 Universality of the Crystallization Protocol

**Figure 6:**
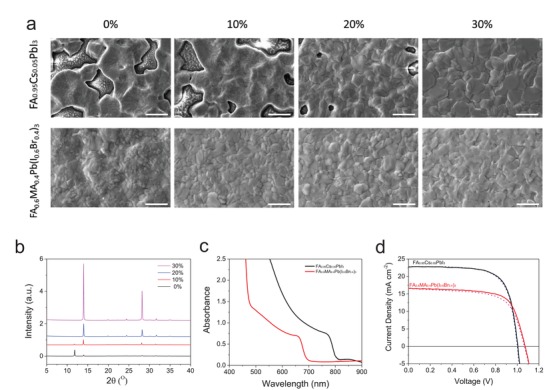
a) SEM images of FA_0.95_Cs_0.05_PbI_3_ films (top row) and FA_0.6_MA_0.4_Pb(I_0.6_Br_0.4_)_3_ films (bottom row) prepared with different levels of MACl additives by blade coating. The scale bars are 2 µm (top row) and 1 µm (bottom row). b) XRD spectra of the FA_0.95_Cs_0.05_PbI_3_ films processed with different MACl contents. c) UV–vis spectra of the FA_0.95_Cs_0.05_PbI_3_ and FA_0.6_MA_0.4_Pb(I_0.6_Br_0.4_)_3_ perovskite films. d) *J–V* curves of the FA_0.95_Cs_0.05_PbI_3_ and FA_0.6_MA_0.4_Pb(I_0.6_Br_0.4_)_3_ perovskite solar cells with both forward (solid line) and reverse scan (dashed line).

Having realized high‐quality perovskite films using MAPbI_3_ as a starting material, we now turn to illustrate the generality of the protocol for other perovskite material systems with particular interest in different band gaps. FAPbI_3_ has a smaller band gap (1.50 eV) than MAPbI_3_ (1.58 eV) and is thus beneficial for increased light harvesting. However, the transition from the α‐phase to the unfavorable yellow δ‐phase at low temperature renders the material not applicable for solar cells. Previous works have shown that addition of 5–20% Cs cations can suppress the formation of the δ‐phase without significantly widening the band gap.^45^, ^46^ We therefore selected FA_0.95_Cs_0.05_PbI_3_ as a smaller band gap material and tested the applicability of our vacuum‐ and additive‐assisted crystallization strategy. Following the same procedure as for the preparation of MAPbI_3_ films, we added different amounts of MACl into the FA_0.95_Cs_0.05_PbI_3_ precursors to facilitate the formation of intermediate phases and examined the morphology and phase of the resulting perovskite films. As shown in **Figure** **6**a, the SEM images reveal an overall crystal enlargement from ≈100 nm to ≈2 µm with increase of MACl additives. The XRD spectra shown in Figure 6b evidences increased characteristic diffraction peaks at 14.01° with higher loads of MACl, which is consistent with the enlarged crystal sizes. Surprisingly, it is found that, without addition of MACl, the presence of Cs alone is incapable of suppressing the formation of δ‐phase (11.81°), as indicated by the coexistence of the yellow δ‐phase and trigonal black FAPbI_3_ α‐phase (14.01°). This observation is strikingly different from the FA_0.95_Cs_0.05_PbI_3_ films prepared by spin‐coating coupled with antisolvent crystallization, where stable α‐phase perovskite films are obtained without any additives.^46^ Remarkably, with incorporation of 10% MACl, the δ‐phase was largely suppressed, concurrent with the formation of a black α‐phase perovskite. Further increasing MACl to 20%, the δ‐phase was eliminated completely and pure α‐phase FA_0.95_Cs_0.05_PbI_3_ was obtained. The suppression of δ‐phase of the FA_0.95_Cs_0.05_PbI_3_ via incorporation of MACl can be presumably due to the formation of intermediate phase that favors the α‐phase formation. To verify this hypothesis, we measured the XRD spectra of the intermediate films with different MACl levels. As shown in Figure S16 of the Supporting Information, the intermediate film without MACl incorporation shows only one peak at 11.81°, indicating the presence of δ‐phase perovskite. Incorporation of 10% MACl yield intermediate film consisting of α‐phase perovskite, intermediate complex, and α‐phase perovskite. This observation suggests that the formation of intermediate complex probably promotes the formation of α‐phase perovskite and simultaneously suppresses the formation of δ‐phase. On further increase of the MACl contents, the δ‐phase was completely eliminated with presence of only the intermediate complex and α‐phase. Overall, these results indeed underline the different crystallization mechanisms of the perovskite films produced by printing techniques compared to those of spin‐coated films, which in turn highlights the necessity to re‐engineer precursor inks as well as crystallization strategies for deposition of perovskite films by scalable methods.

Figure 6c presents the UV–vis absorption of thin film FA_0.95_Cs_0.05_PbI_3_ which clearly evidences a reduced band gap compared to MAPbI_3_. We prepared solar cells using perovskite films processed with 30% MACl additives to evaluate the photovoltaic performance of the blade‐deposited FA_0.95_Cs_0.05_PbI_3_ films. The best solar cell shows a *J*_SC_ of 22.81, a *V*_OC_ of 1.01 V, and an FF of 72.20% yielding a decent PCE of 16.63% with a negligible hysteresis (Figure 6d). The EQE response of the solar cell extends to 850 nm, giving an integrated current density of 21.85 mA cm^−2^ (Figure S17, Supporting Information).

We continue to demonstrate the versatility of our method by fabricating a wide‐band gap perovskite composite. Wider band gap perovskites are in the research focus for multijunction photovoltaics. Partial substitution of iodine with bromide can effectively broaden the band gap of the perovskites. We thus prepared mixed‐halide perovskite films with a formula of FA_0.6_MA_0.4_Pb(I_0.6_Br_0.4_)_3_, which has an optical band of 1.76 eV (Figure 6c and Figure S17, Supporting Information). As expected, SEM images (Figure 6a) and XRD patterns of the prepared films (Figure S18, Supporting Information) once again confirmed enhanced crystal size and crystallinity with addition of MACl. The best solar cell fabricated from 10% MACl additive yields a high *V*_OC_ of 1.07 V, FF of 69.50%, and a *J*_SC_ of 16.59 mA cm^−2^, giving a PCE of 12.34% with a small hysteresis. The relatively low current density is mainly related to the narrow solar spectrum coverage due to its wide band gap (Figure S17, Supporting Information).

## 3 Conclusion

In summary, we have developed a generic and robust crystallization technology specifically targeted at scalable deposition of high‐quality perovskite films via printing methods. In contrast to previously proposed strategies with which the crystallization was directly driven by applying high temperatures during precursor deposition, the essence of our technology is the deployment of a vacuum‐based precrystallization procedure. The vacuum extraction produces temporary intermediate films, allowing the precursor film deposition to be separated from the subsequent postannealing. Equally important, the incorporation of small amounts of MACl in precursor inks promotes the formation of intermediate films, which enables us to precisely control the morphology, phase, and crystallinity of perovskite films over a wide range of compositions. Multiple‐cation and mixed halide perovskite composites with different band gaps have successfully been fabricated, attesting the general application of the technology for perovskite thin film processing. From the perspective of the global photovoltaics community, these encouraging results are anticipated to inspire the field to make a significant step toward the reproducible manufacturing of wafer‐scale perovskite modules by high‐throughput printing methods.

## 4 Experimental Section

*Materials*: Lead iodide (PbI_2_, 99.9985%) and cesium iodide (CsI, 99.999%) were purchased from Alfa Aesar. Lead bromide (PbBr_2_, 99.999%), Methylammonium chloride (MACl, 98%), and all the solvents were purchased from Sigma‐Aldrich. Methylammonium iodide (MAI), Methylammonium bromide (MABr), and Poly[bis(4‐phenyl)(2,4,6‐trimethylphenyl)amine] (PTAA) were purchased from Xi'an p‐OLED Co. (China). Ammonium thiocyanate (NH_4_SCN, 98%) and potassium thiocyanate (KSCN, 99.99%) were purchased from Tokyo Chemical Industry (TCI) and Aladdin, respectively. Bathocuproine (BCP), formamidinium iodide (FAI), PC_61_BM was purchased from Lumtec. All the chemicals were used as received without further purification.

*Blade Deposition of Perovskite Films*: For MAPbI_3_ material system, the precursor solution was prepared by dissolving 1 m equimolar ratio of PbI_2_ and MAI with different amounts of additives (MACl: 10%, 20%, 30%, 50%, 100% mole ratio; the mole ratio for NH_4_SCN and KSCN is 5%, 10%, 30%) in mixed solvent of DMF and DMSO (volume ratio 4:1). Blade coating of the perovskite precursor films was carried out on a commercial blade coater (ZAA2300.H from ZEHNTNER) using a ZUA 2000.100 blade (from ZEHNTNER) at room temperature in nitrogen‐filled glovebox. For the substrate with dimension of 25 × 25 mm, 20 µL solution was used for blade deposition. The gap for solution load between the substrate and blade was fixed at 200 µm. Once the precursor solution spread onto the substrate by blade‐coating, the liquid precursor film was transferred into a vacuum chamber, which was pumped to 1000 Pa in 15 s and stayed at the pressure for 2 min. During the vacuum process at around 1 min, the transparent yellow liquid film turned to brownish, indicating the formation of an intermediate film. The obtained intermediate film was subsequently brought out of the vacuum chamber and annealed at 100 °C for 10 min in the glovebox to fully crystallize the film.

For the blade‐deposition of FA_0.95_Cs_0.05_PbI_3_ and FA_0.6_MA_0.4_Pb(I_0.6_Br_0.4_)_3_ material systems, the 1m precursor solution were prepared as follows. FA_0.95_Cs_0.05_PbI_3_: 1 mmol PbI_2_, 0.95 mmol FAI, and 0.05 mmol CsI; FA_0.6_MA_0.4_Pb(I_0.6_Br_0.4_)_3_: 0.6 mmol PbI_2_, 0.4 mmol PbBr_2_, 0.6 mmol FAI, and 0.4 mmol MABr. Similarly, the 10%, 20%, and 30% ratio of MACl was added into the 1m mother solutions to obtain the respective FA_0.95_Cs_0.05_PbI_3_ and FA_0.6_MA_0.4_Pb(I_0.6_Br_0.4_)_3_ precursor inks for blade coating. The perovskite films were prepared by blade coating following the same procedure for the deposition of MAPbI_3_. The annealing temperature for FA_0.95_Cs_0.05_PbI_3_ and FA_0.6_MA_0.4_Pb(I_0.6_Br_0.4_)_3_ was increased to 140 °C owing to the presence of FA in the system which requires higher temperature to convert the intermediate phase into perovskites.

*Solar Cell Fabrication*: The prepatterned indium tin oxide (ITO) coated glass (OPV Tech Co., Ltd.) was sequentially cleaned by sonicating the substrates in acetone and isopropanol for 10 min each. A PTAA hole‐transporting layer was spin‐coated from 2.5 mg mL^−1^ CB solution on ITO substrate at 5000 rpm for 30 s. The film was then annealed at 120 °C for 5 min in ambient air. The substrate was transferred to a nitrogen‐filled glovebox after it cooled down to room temperature. The perovskite absorber layer was subsequently deposited using the vacuum‐assisted blade‐coating method as described above. On top of perovskite film, the electrotransporting layer PC_61_BM (20 mg mL^−1^ in chlorobenzene) and the interfacial layer BCP (2.5 mg mL^−1^ in isopropanol) was successively deposited by spin coating at 2000 rpm for 30 s and 5000 rpm for 30 s, respectively. Finally, 100 nm Ag contact was deposited by thermal evaporation. The active areas of the solar cells were 0.09 and 1 cm^2^, respectively, for the small‐size (MAPbI_3_, FA_0.95_Cs_0.05_PbI_3_, and FA_0.6_MA_0.4_Pb(I_0.6_Br_0.4_)_3_) and large‐area (MAPbI_3_) devices, which were determined by the overlapping between the top Ag and bottom ITO electrode. All the devices for performance and stability evaluation were tested without encapsulation.

Large‐area MAPbI_3_ modules with four cells monolithic interconnected in series were fabricated on 4 × 4 cm^2^ ITO‐coated glass substrates. The P1 lines on ITO substrates with a space of 8 mm are prepatterned by laser scribing. On the cleaned ITO substrates, the hole‐transporting layer PTAA was deposited by spin coating at 5000 rpm for 30 s. The perovskite film was then blade coated as detailed above. On top of perovskite layer, the electron‐transporting layer PC_61_BM and buffer layer BCP were sequentially deposited by spin coating at 2500 rpm. for 35 s and 5000 rpm for 1 min, respectively. Three P2 lines were scribed mechanically using a tweezer parallel to the P1 lines. To complete the module fabrication, 100 nm Ag was thermally deposited using a shadow mask which was prepatterned to function the P3 lines. The series‐interconnection of the module was realized by the mechanically separated but electrically interconnected P1, P2, and P3 lines (Figure S15, Supporting Information).

*Characterizations*: Morphologies of the perovskite films were imaged with a scanning electron microscope (SEM, FEI Apreo LoVac). The structural properties of the perovskite films were analyzed by a Bruker D8 Advance X‐ray diffractometer (XRD) using Cu Kα as the radiation source. The X‐ray photoelectron spectroscopy (XPS) measurements were performed on an ESCALab250Xi electron spectrometer (Thermo Fisher) using Al Kα radiation. Transmittance Infrared (IR) measurements were carried out employing an FTIR spectrometer (Thermo Scientific Nicolet 380). Optical absorption measurements were performed on a Agilent Cary 5000 spectrophotometer. The Tindall effect was tested with a commercial laser pen with green beam light. The current density–voltage (*J–V*) characteristics and steady‐state output of all the solar cells were measured using a Keithley 2400 source meter. The illumination was provided by a Newport Oriel 92 192 solar simulator with an AM1.5G filter, operating at 100 mW cm^−2^, which was calibrated by a standard silicon solar cell from Newport. The EQE was taken using a QE‐R instrument from Enlitech.

## Conflict of Interest

The authors declare no conflict of interest.

## Supporting information

Supplementary
