# Supplementary material for: A Generalized Crystallization Protocol for Scalable Deposition of High‐Quality Perovskite Thin Films for Photovoltaic Applications
Source: Adv Sci (Weinh). 2019 Jun 25;6(17):1901067. doi: 10.1002/advs.201901067 (PMC6724353; doi:10.1002/advs.201901067)
Supplement: Supplementary file 1 — Supplementary [file ADVS-6-1901067-s001.pdf]

Copyright WILEY-VCH Verlag GmbH & Co. KGaA, 69469 Weinheim, Germany, 2019.

Supporting Information

for *Adv. Sci.,* DOI: 10.1002/advs.201901067

A Generalized Crystallization Protocol for Scalable Deposition of High-Quality Perovskite Thin Films for Photovoltaic Applications

Fei Guo, Shudi Qiu, Jinlong Hu, Huahua Wang, Boyuan Cai,* Jianjun Li, Xiaocong Yuan, Xianhu Liu, Karen Forberich, Christoph J. Brabec, and Yaohua Mai**

Supplemental Information

A generalized crystallization protocol for scalable deposition of high-quality perovskite thin films for photovoltaic applications

Fei Guo[1‡], Shudi Qiu[1‡], Jinlong Hu[1], Huahua Wang[2], Boyuan Cai[2*], jianjun Li[1], Xiaocong Yuan[2], Xianhu Liu[3], Karen Forberich[4], Christoph J. Brabec[4] and Yaohua Mai[*1]*

[1]Institute of New Energy Technology, College of Information Science and Technology, Jinan University, Guangzhou 510632, China. *E-mail: fei.guo@jnu.edu.cn; yaohuamai@jnu.edu.cn*

[2]Nanophotonics Research Center, Shenzhen Key Laboratory of Micro-scale Optical Information Technology, Shenzhen University, Shenzhen 518060, China. *E-mail: caiboyuan@szu.edu.cn*

[3]National Engineering Research Center for Advanced Polymer Processing Technology, Zhengzhou University, Zhengzhou, 450002 China

[4]Institute of Materials for Electronics and Energy Technology (i-MEET), Friedrich-Alexander University Erlangen-Nürnberg, Martensstrasse 7, 91058 Erlangen, Germany.

[‡]These authors contributed equally to this work.

Table S1. The efficiencies of the perovskite solar cells deposited by scalable printing methods collected from the previous reports with comparison to the present work.

References	Efficiency (%)	Deposition Temperature (°C)	Device area	Note
[1]	18.55	25	0.12 cm^2	Blade coating
[2]	**18.06**	**25**	0.09 cm^2	Blade coating (**Our work**)
[3]	11.32	25	0.1 cm^2	Blade coating
[4]	20.05	60	0.1 cm^2	Meniscus-assisted blade-coating
[5]	15.57	60	0.1 cm^2	Slot-die coating
[6]	11.96	70	0.1 cm^2	Slot-die coated
[7]	9.2	65	0.0625	Slot-die coating
[8]	20.2	100	0.08	Blade coating
[9]	17.6	95	1 cm^2	Soft-cover deposition
[10]	16.9	100	-	Blade coating
[11]	16	100	1.2 cm^2	Spray coating
[12]	12.2	100	-	Blade coating
[13]	19.2	120	-	Blade coating
[14]	15.1	125	1 cm^2	Slot-die coating
[15]	10.92	130	0.09 cm^2	Blade coating
[16]	20.3	145	7.5 mm^2	Blade coating
[17]	18.74	150	0.09	Blade coating
[18]	17.54	150	-	Blade coating
[19]	16.01	150	0.11 cm^2	Blade coating
[20]	15.8	135	0.1 cm^2	Blade coating
[21]	14.4	140	0.1 cm^2	Slot-die coating
[22]	7.32	150	1 cm^2	Blade coating

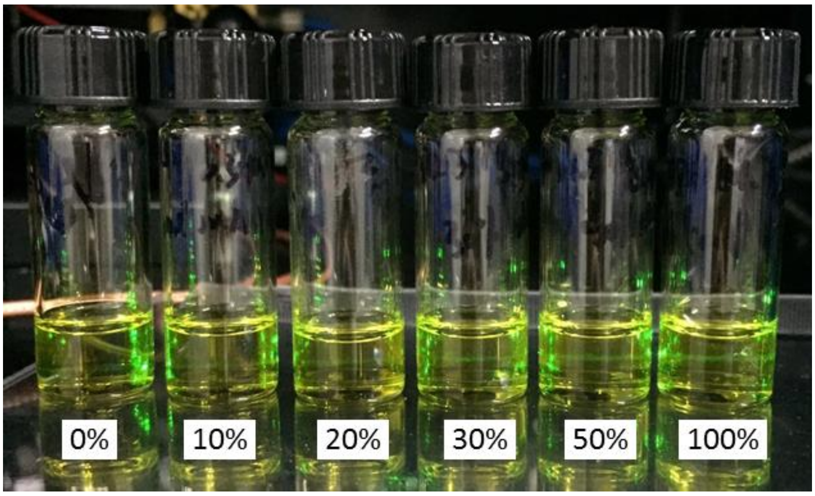

Figure S1. Tindall test of $MAPbI_3$ precursor solutions with 0~100% MACl additives. It is noticed that the higher the MACl additive the stronger the Tindall effect are observed, suggesting that the size of aggregated colloid particles increases proportional to the MACl content.

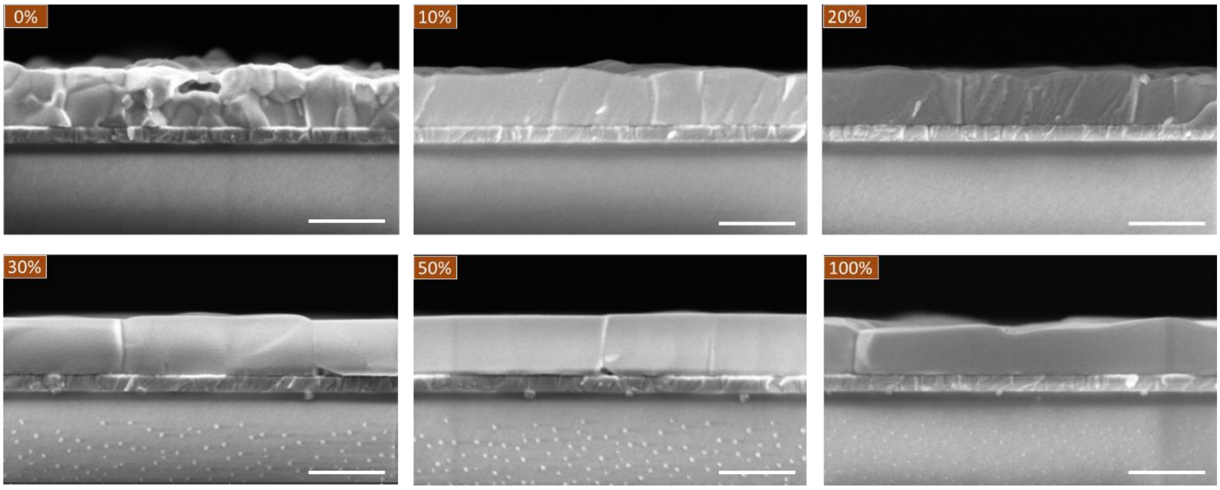

Figure S2. Cross sectional SEM images of the blade-coated perovskite films prepared from the precursor inks with different amounts of MACl additives. The scale bars are 500 nm.

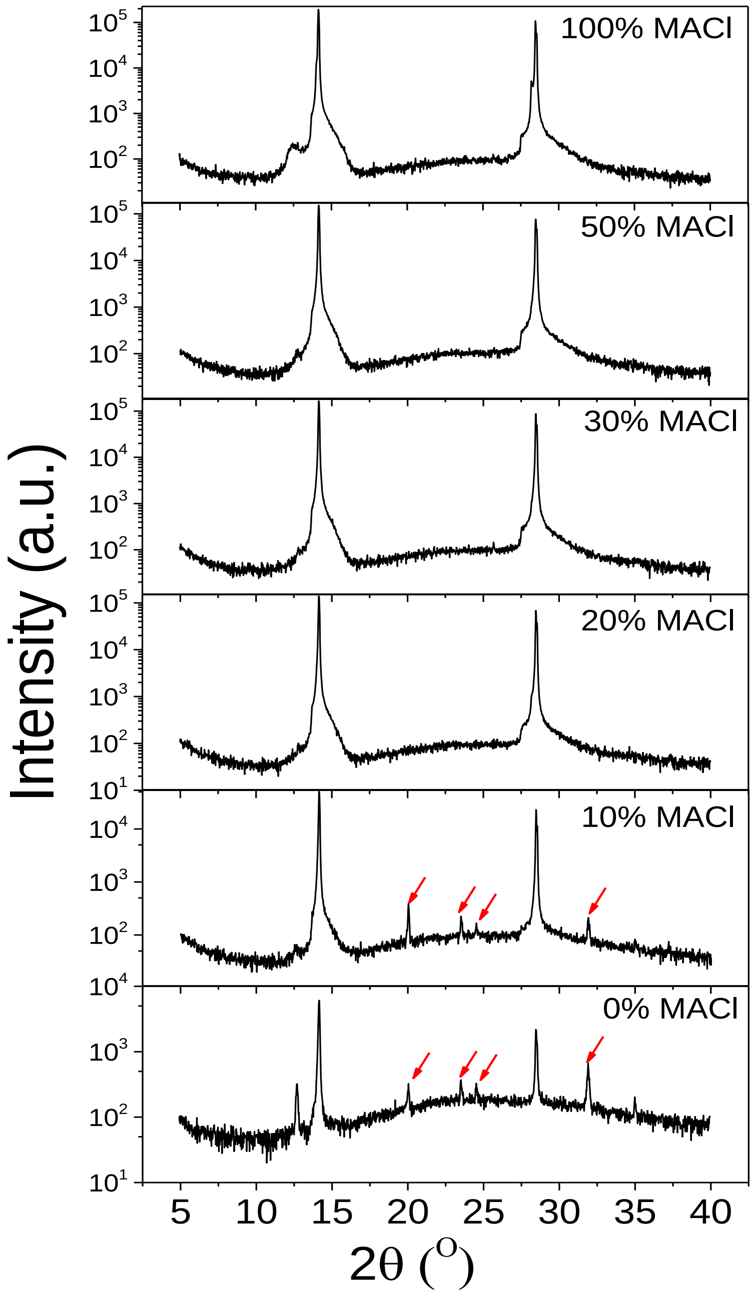

Figure S3. XRD patterns of blade-coated perovskite films with different amount of MACl. The figure was replotted from the **Figure 3d** with the Y axis in logarithm sale.

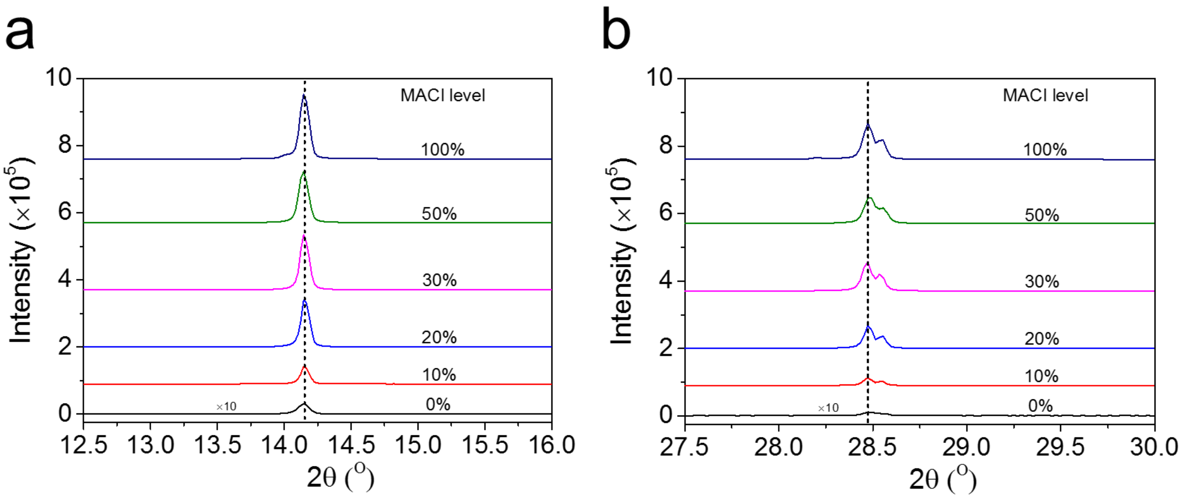

Figure S4. Zoom-in spectra of the (110) and (220) planes of the XRD of the $MAPbI_3$ film with different levels of MACl additives.

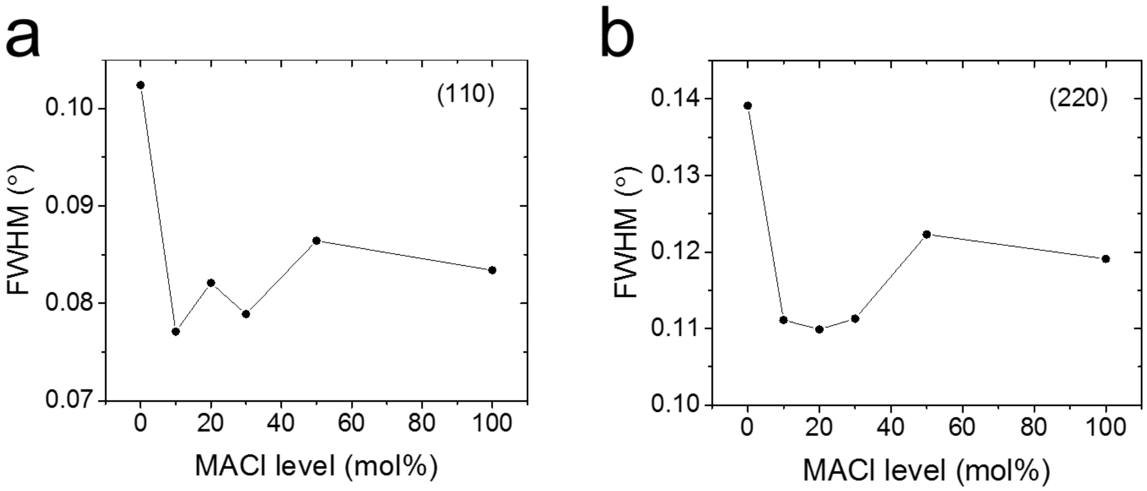

Figure S5. Full width at half maximum (FWHM) of the (110) and (220) peaks of $MAPbI_3$ films as a function of MACl addition level.

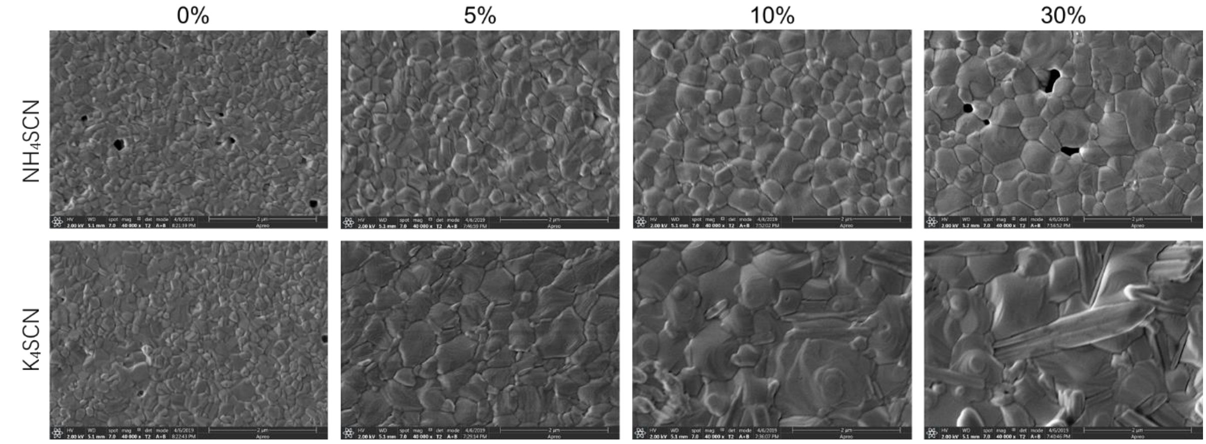

Figure S6. Top-view SEM images of the blade-coated $MAPbI_3$ films processed with different amounts of additives of NH_4SCN and KSCN.

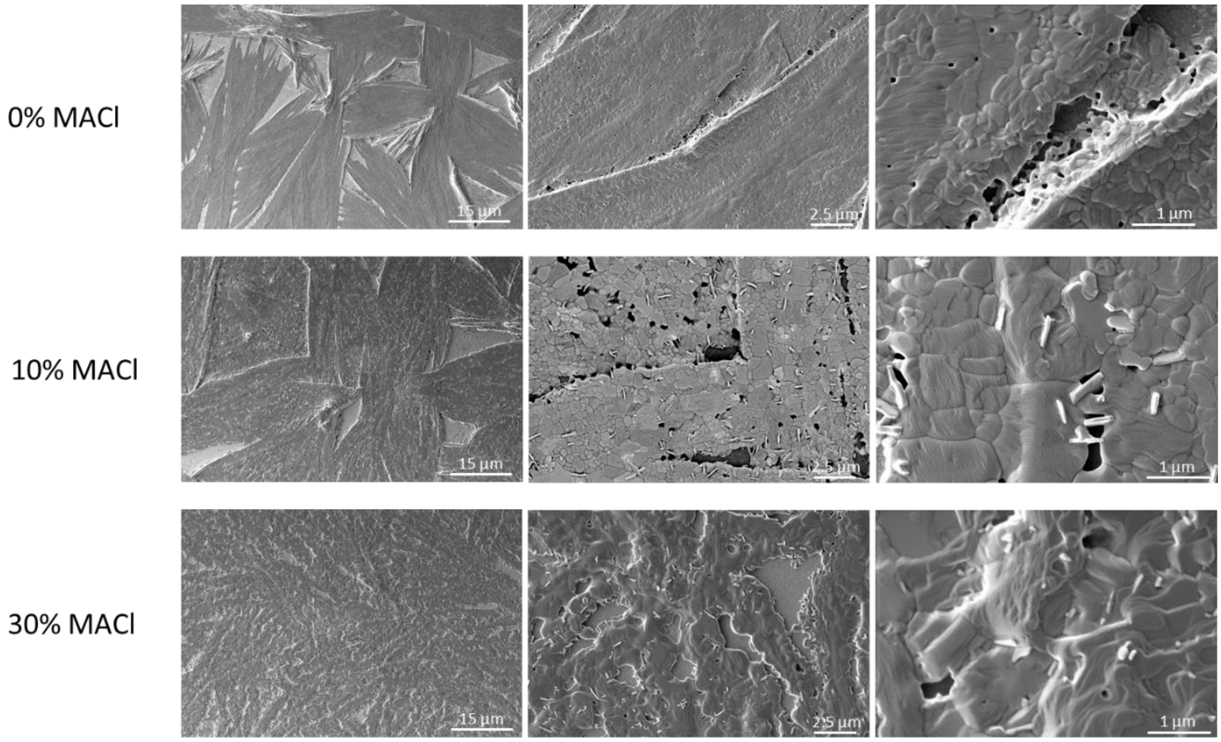

Figure S7. Top-view SEM images of the blade-deposited $MAPbI_3$ films processed with different levels of MACl additive obtained by thermal annealing of the naturally precursor film (without vacuum exaction process).

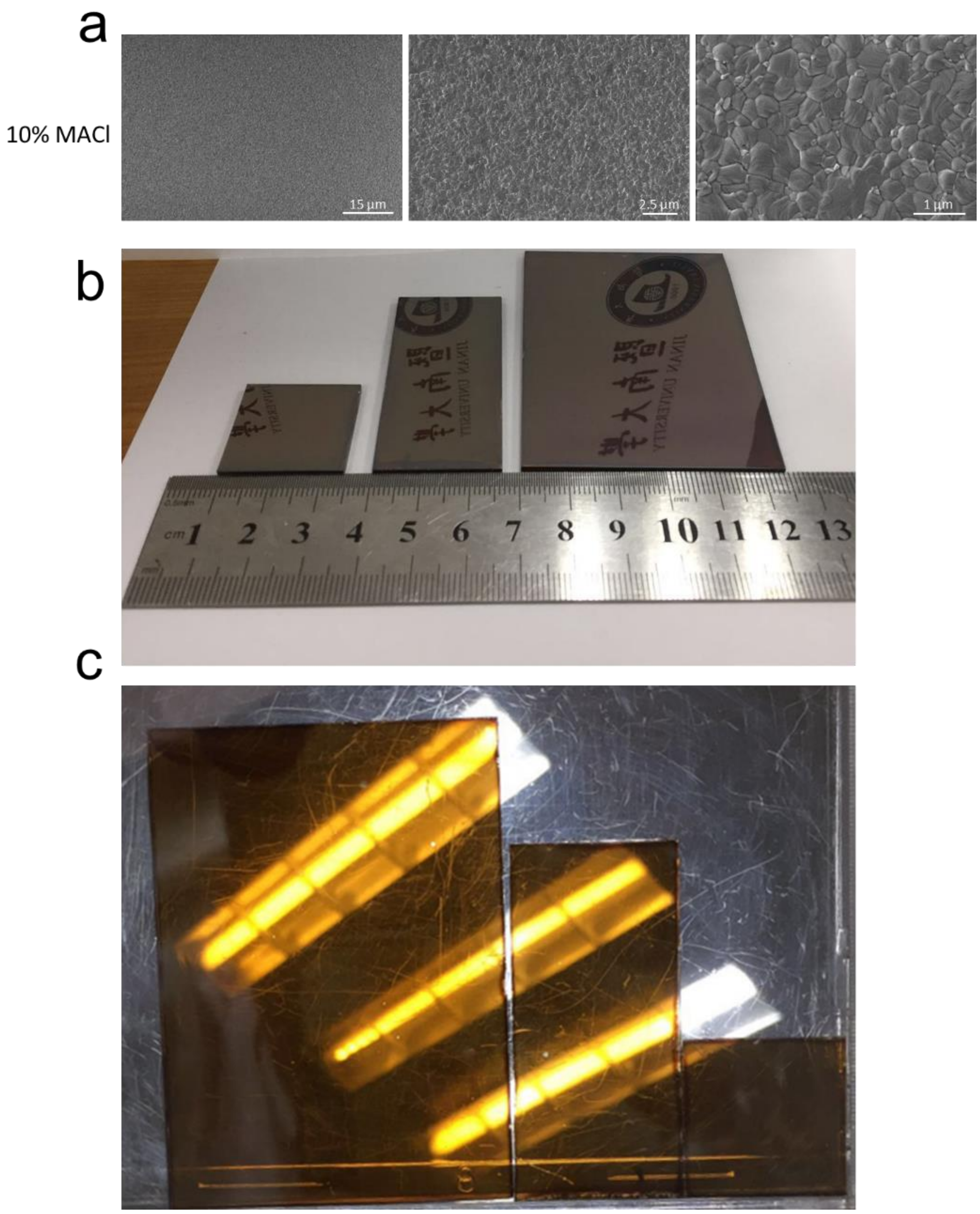

Figure S8. (a) Top-view SEM images in different magnifications of the blade-deposited $MAPbI_3$ films processed from 10% MACl additive, which was prepared by vacuum-assisted crystallization method. (b, c) Digital images of the $MAPbI_3$ films with dimensions of 2.5×2.5 cm^2, 2.5×5 cm^2 and 5.5×7 cm^2, which shows high reflectance (**b**) and non-scattered transmittance (**c**).

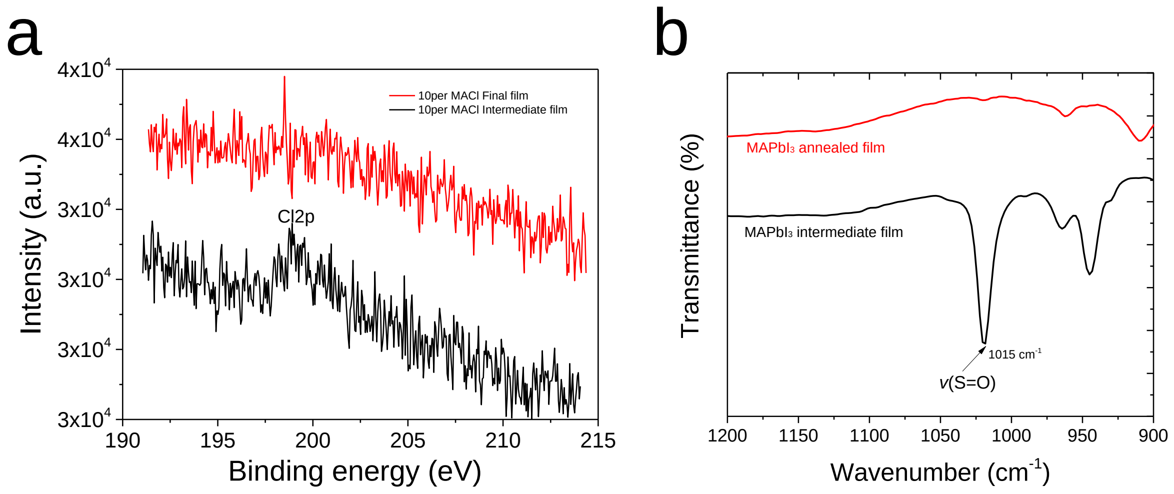

Figure S9. (a) XPS spectra of the Cl element scanned from intermediate film and final crystalline $MAPbI_3$ film prepared with 10% MACl additive. (b) FTIR spectrum of the intermediate $MAPbI_3$ film prepared with 10% MACl additive.

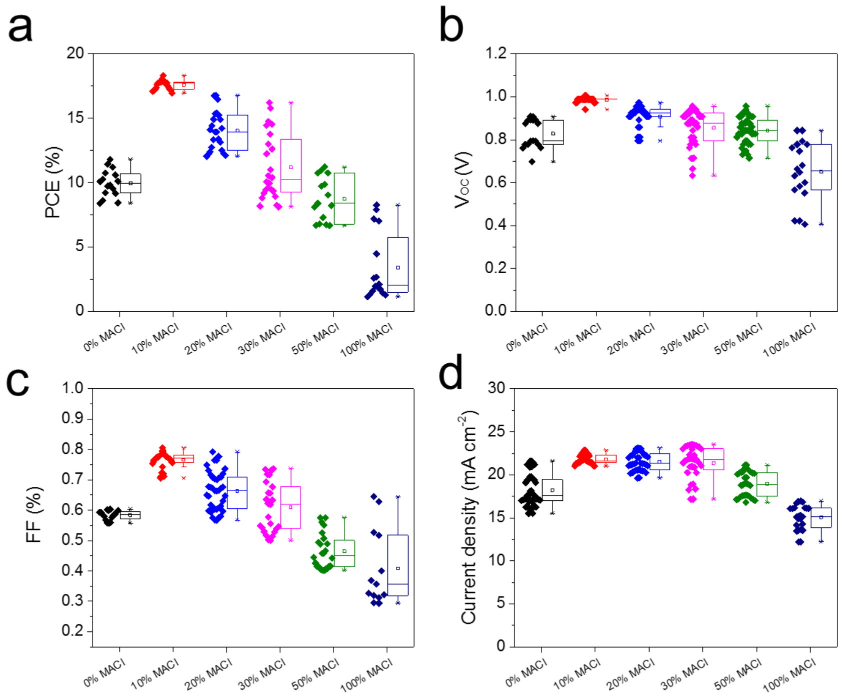

Figure S10. Device performance of the doctor-blade coated $MAPbI_3$ solar cells prepared from MACl additives with molar ratios of 0, 10%, 20%, 30%, 50%, 100%: (a) PCE, (b) V_{OC}, (c) FF, (d) J_{SC}.

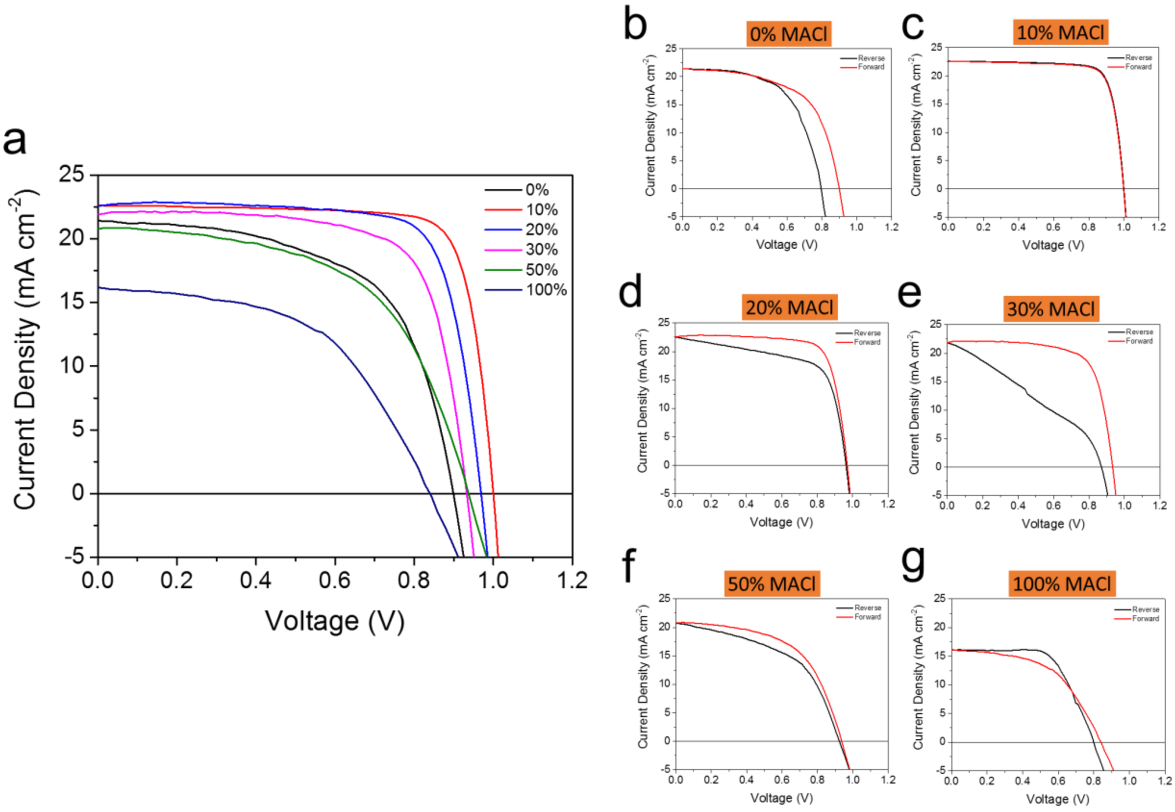

Figure S11. (a) J-V curves of the $MAPbI_3$ solar cells with different molar ratios of MACl (forward scan). (b)-(g) J-V curves of the solar cells with different amount of MACl additives measured from both reverse and forward scans.

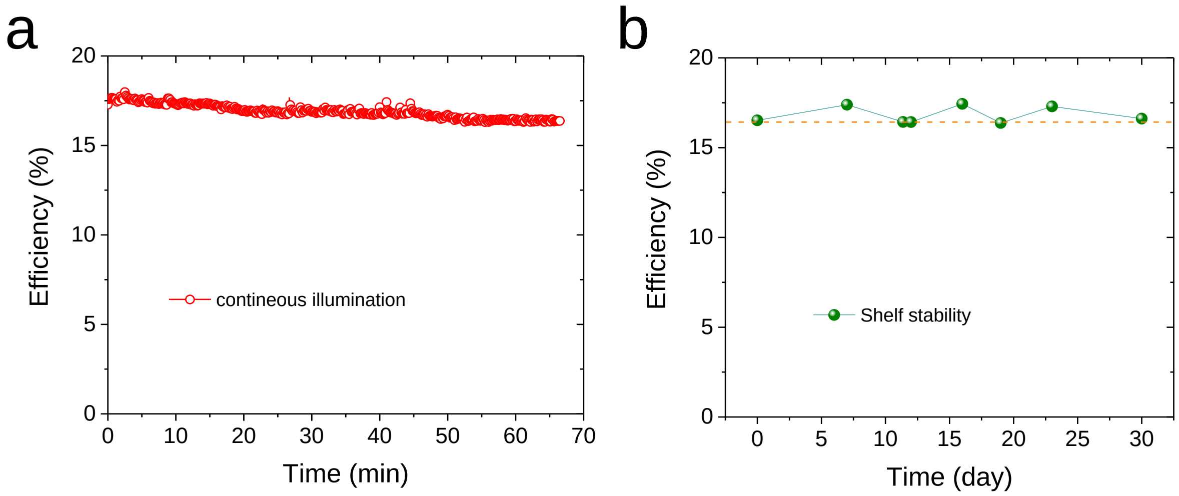

Figure S12. (a) The efficiency evolution of the $MAPbI_3$ solar cells under continuous AM 1.5G illumination with intensity of 100 mW cm^{-2} for 66 minutes. (b) Shelf stability of a $MAPbI_3$ solar cell stored in glovebox for 30 days.

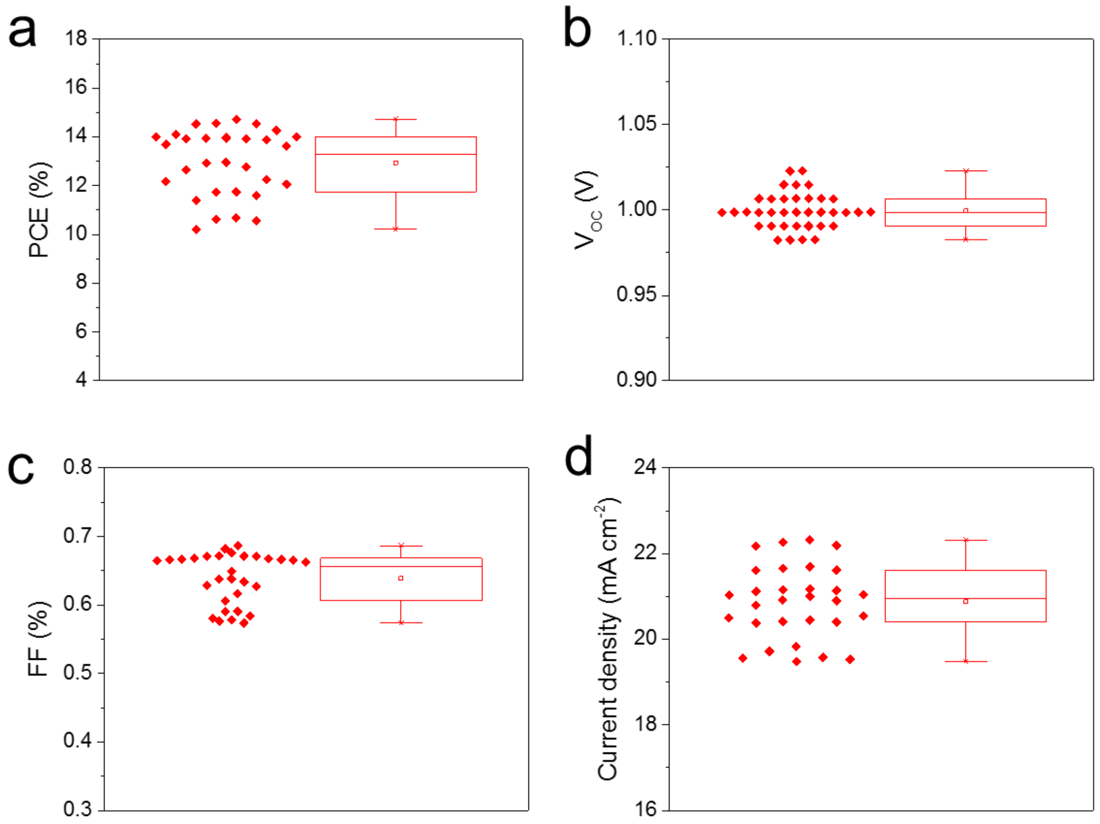

Figure S13. Statistics of PCE (a), V_{OC} (b), FF (c), and J_{SC} (d) of 15 $MAPbI_3$ solar cells (10% MACl additives) with active area of 1 cm^2 measured from both forward and reverse scan directions.

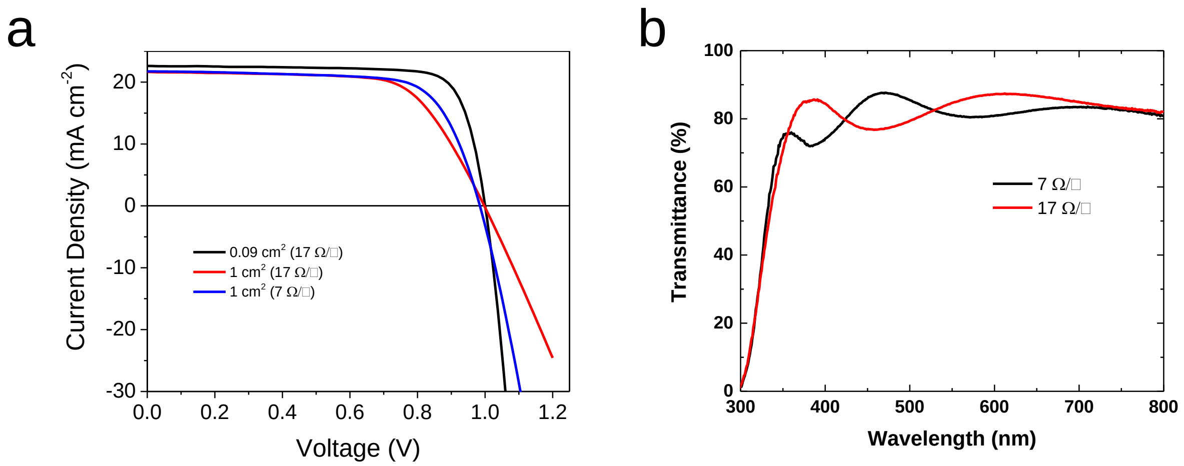

Figure S14. (a) J-V curves of the best-performing blade-coated $MAPbI_3$ solar cells deposited on ITO with different sheet resistances and with different active areas. (b) UV-vis transmittance of the two types of ITO electrodes with different sheet resistances.

Table S2. Photovoltaic parameters of the best-performing blade-coated $MAPbI_3$ solar cells deposited on ITO with different sheet resistances and with different active areas.

**Device area (cm^2)**	**ITO sheet resistance [Ω/sq]**	**V_{OC} [V]**	**FF [%]**	**J_{SC} [mA/cm^2]**	**PCE [%]**	**R_P [Ω cm^2]**	**R_S [Ω cm^2]**
**0.09**	**17**	1.00	80.00	22.58	18.06	2037	**1.85**
**1**	**17**	1.01	67.15	21.70	14.72	1376	**8.25**
**1**	**7**	0.99	72.60	21.73	15.62	1043	**3.89**

Note: Parallel resistance (R_P) and series resistance (R_S) are calculated from the J-V curves shown in **Figure S14** in the voltage range of 0-0.2 V and 1-1.1V, respectively.

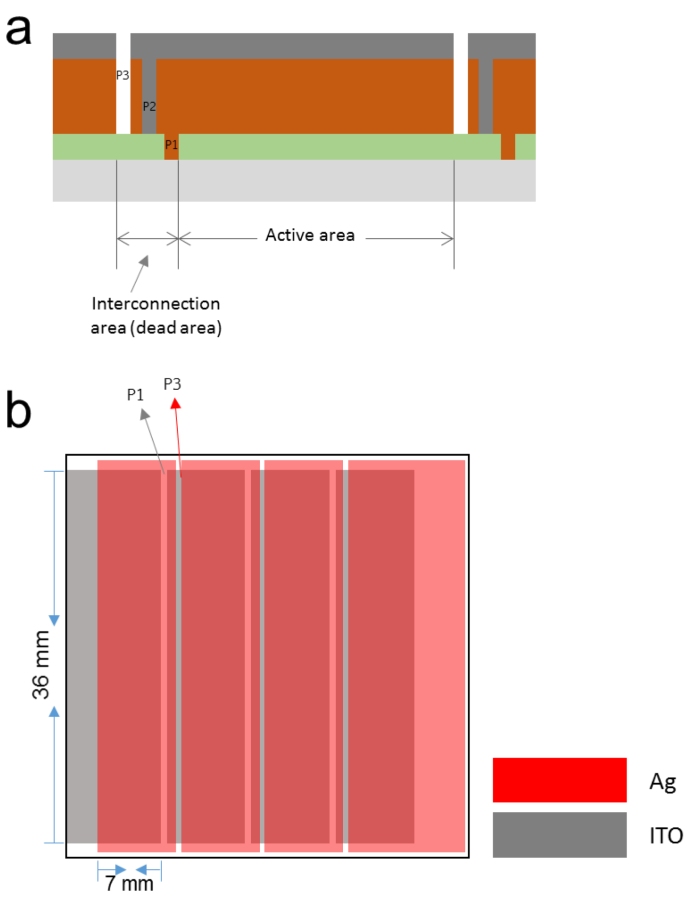

Figure S15. Module interconnection illustration. (a) Schematic of module interconnection with P1, P2, P3 scribing. (b) Top-view illustration of the module where the length and width of the individual subcell is 3.6 cm and 0.7 cm respectively, giving the total active area of the module of 10.08 cm^2. The width of the interconnection area is around 2 mm, which gives a non-working area (dead area) of 2.88 cm^2.

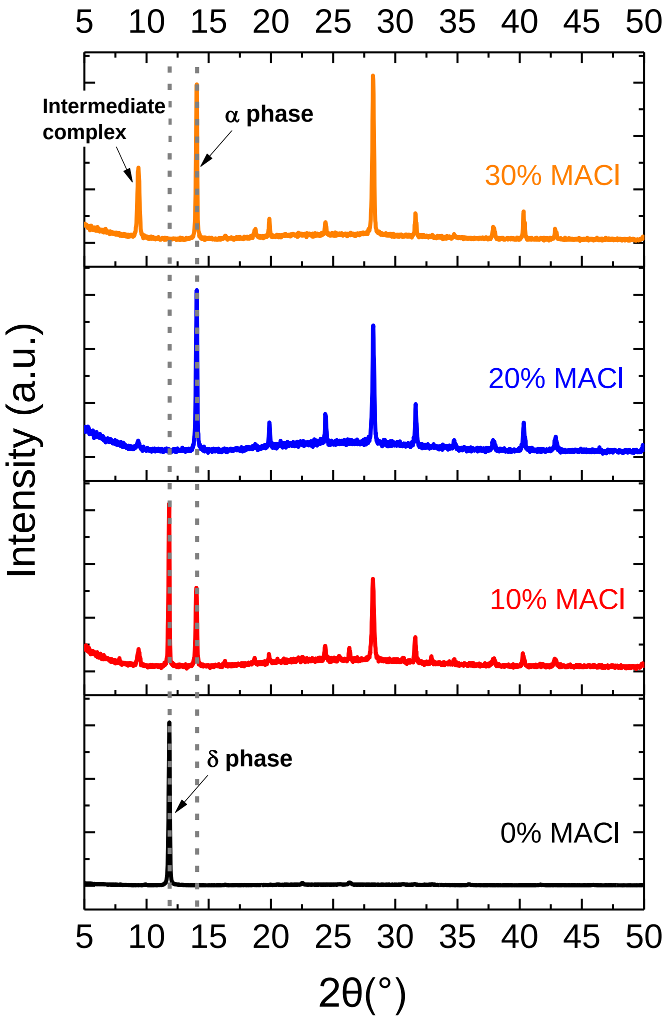

Figure S16. XRD spectra of the intermediate films of $FA_{0.95}Cs_{0.05}PbI_3$ processed with different levels of MACl additives.

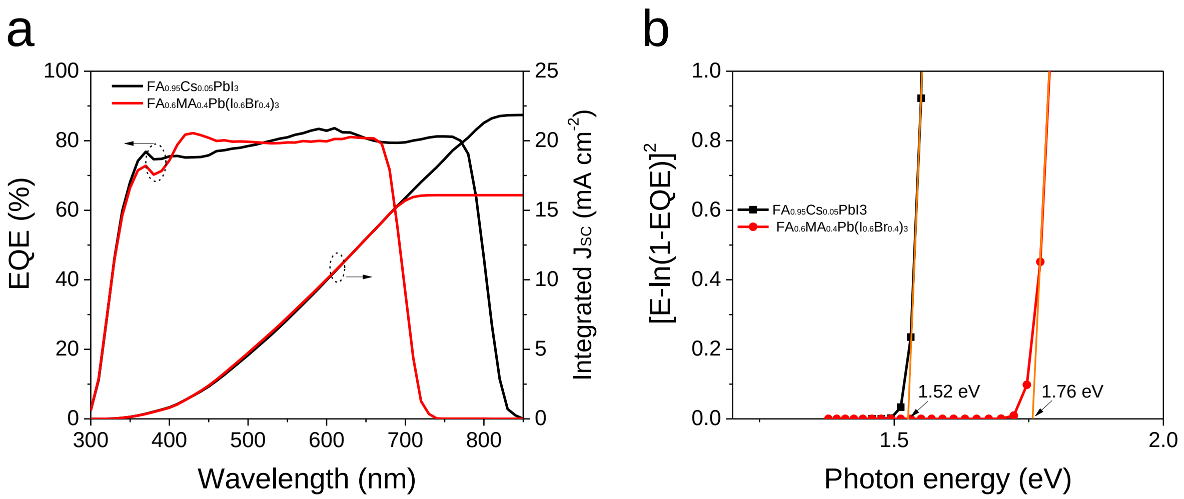

Figure S17. (a) EQE spectra of the $FA_{0.95}Cs_{0.05}PbI_3$ and $FA_{0.6}MA_{0.4}Pb(I_{0.6}Br_{0.4})_3$ perovskite solar cells, which give integrated current densities of 21.85 mA cm^{-2} and 16.09 mA cm^{-2}, respectively. (b) Band gap (E_g) of the two absorbers derived from the EQE.

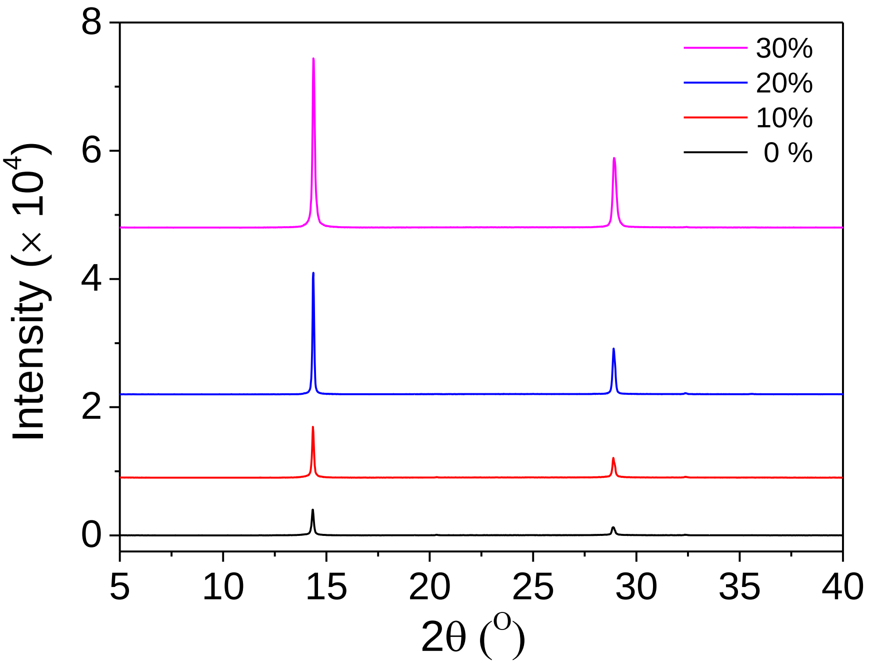

Figure S18. XRD spectra of the $FA_{0.6}MA_{0.4}Pb(I_{0.6}Br_{0.4})_3$ films processed with different MACl contents.

References:

1. Yang, M. J.; Li, Z.; Reese, M. O.; Reid, O. G.; Kim, D. H.; Siol, S.; Klein, T. R.; Yan, Y.; Berry, J. J.; van Hest, M. F. A. M.; Zhu, K., Perovskite ink with wide processing window for scalable high-efficiency solar cells. *Nat Energy* **2017**, 2 (5). [Note: The perovskite precursor was bladed at room-temperature, while the crystallization was conducted in an anti-solvent bath.]

2. **Our work.** [Note: The perovskite precursor was bladed at room-temperature, followed by vacuum extraction to achieve a stable supersaturation stage, which decouples the precursor deposition and the subsequent annealing-based crystallization.]

3. Yang, Z. B.; Chueh, C. C.; Zuo, F.; Kim, J. H.; Liang, P. W.; Jen, A. K. Y., High-Performance Fully Printable Perovskite Solar Cells via Blade-Coating Technique under the Ambient Condition. *Adv Energy Mater* **2015**, 5 (13). [Note: Typical procedure for the perovskite precursor deposition at room-temperatue which was followed by an annealing-based crystallization.]

4. He, M.; Li, B.; Cui, X.; Jiang, B. B.; He, Y. J.; Chen, Y. H.; O'Neil, D.; Szymanski, P.; El-Sayed, M. A.; Huang, J. S.; Lin, Z. Q., Meniscus-assisted solution printing of large-grained perovskite films for high-efficiency solar cells. *Nat Commun* **2017**, 8. [Note: The perovskite precursor was deposited at elevated temperature of 60 °C, but the blade speed was rather slow (12 μm/s).]

5. Zuo, C. T.; Vak, D.; Angmo, D.; Ding, L. M.; Gao, M., One-step roll-to-roll air processed high efficiency perovskite solar cells. *Nano Energy* **2018**, 46, 185-192. [Note: N_2 blowing assisted perovskite deposition at 60 °C.]

6. Hwang, K.; Jung, Y. S.; Heo, Y. J.; Scholes, F. H.; Watkins, S. E.; Subbiah, J.; Jones, D. J.; Kim, D. Y.; Vak, D., Toward Large Scale Roll-to-Roll Production of Fully Printed Perovskite Solar Cells. *Adv Mater* **2015**, 27 (7), 1241-1247. [Note: N_2 blowing assisted perovskite deposition at 70 °C.]

7. Cotella, G.; Baker, J.; Worsley, D.; De Rossi, F.; Pleydell-Pearce, C.; Carnie, M.; Watson, T., One-step deposition by slot-die coating of mixed lead halide perovskite for photovoltaic applications. *Sol Energ Mat Sol C* **2017**, 159, 362-369. [Note: N_2 blowing assisted perovskite deposition at 65 °C.]

8. Wu, W. Q.; Wang, Q.; Fang, Y. J.; Shao, Y. C.; Tang, S.; Deng, Y. H.; Lu, H. D.; Liu, Y.; Li, T.; Yang, Z. B.; Gruverman, A.; Huang, J. S., Molecular doping enabled scalable blading of efficient hole-transport-layer-free perovskite solar cells. *Nat Commun* **2018**, 9. [Note: Simultaneous solvent evaporation and crystallization with precursor perovskite deposition at 100 °C.]

9. Ye, F.; Chen, H.; Xie, F. X.; Tang, W. T.; Yin, M. S.; He, J. J.; Bi, E. B.; Wang, Y. B.; Yang, X. D.; Han, L. Y., Soft-cover deposition of scaling-up uniform perovskite thin films for high cost-performance solar cells. *Energ Environ Sci* **2016**, 9 (7), 2295-2301. [Note: Soft-cover technique with the simultaneous solvent evaporation and crystallization at 90 °C.]

10. Deng, Y. H.; Dong, Q. F.; Bi, C.; Yuan, Y. B.; Huang, J. S., Air-Stable, Efficient Mixed-Cation Perovskite Solar Cells with Cu Electrode by Scalable Fabrication of Active Layer. *Adv Energy Mater* **2016**, 6 (11). [Note: Simultaneous solvent evaporation and crystallization with precursor perovskite deposition at 100 °C.]

11. Kim, J.; Yun, J. S.; Cho, Y.; Lee, D. S.; Wilkinson, B.; Soufiani, A. M.; Deng, X. F.; Zheng, J. H.; Shi, A.; Lim, S.; Chen, S.; Hameiri, Z.; Zhang, M.; Lau, C. F. J.; Huang, S. J.; Green, M. A.; Ho-Baillie, A. W. Y., Overcoming the Challenges of Large-Area High-Efficiency Perovskite Solar Cells. *Acs Energy Lett* **2017**, 2 (9), 1978-1984. [Note: Spray antisolvent crystallization with precursor perovskite deposition at 100 °C.]

12. Deng, Y. H.; Wang, Q.; Yuan, Y. B.; Huang, J. S., Vividly colorful hybrid perovskite solar cells by doctor-blade coating with perovskite photonic nanostructures. *Mater Horiz* **2015**, 2 (6), 578-583. [Note: Simultaneous solvent evaporation and crystallization with precursor perovskite deposition at 100 °C.]

13. Tang, S.; Deng, Y. H.; Zheng, X. P.; Bai, Y.; Fang, Y. J.; Dong, Q. F.; Wei, H. T.; Huang, J. S., Composition Engineering in Doctor-Blading of Perovskite Solar Cells. *Adv Energy Mater* **2017**, 7 (18). [Note: Simultaneous solvent evaporation and crystallization with precursor deposited at 120 °C.]

14. Deng, Y. H.; Peng, E.; Shao, Y. C.; Xiao, Z. G.; Dong, Q. F.; Huang, J. S., Scalable fabrication of efficient organolead trihalide perovskite solar cells with doctor-bladed active layers. *Energ Environ Sci* **2015**, 8 (5), 1544-1550. [Note: Simultaneous solvent evaporation and crystallization with precursor deposited at 100 °C.]

15. Hilt, F.; Hovish, M. Q.; Rolston, N.; Bruning, K.; Tassone, C. J.; Dauskardt, R. H., Rapid route to efficient, scalable, and robust perovskite photovoltaics in air. *Energ Environ Sci* **2018**, 11 (8), 2102-2113.

16. Deng, Y. H.; Zheng, X. P.; Bai, Y.; Wang, Q.; Zhao, J. J.; Huang, J. S., Surfactant-controlled ink drying enables high-speed deposition of perovskite films for efficient photovoltaic modules. *Nat Energy* **2018**, 3 (7), 560-566. [Note: Simultaneous solvent evaporation and crystallization with precursor deposited at 120 °C.]

17. Li, J. B.; Munir, R.; Fan, Y. Y.; Niu, T. Q.; Liu, Y. C.; Zhong, Y. F.; Yang, Z.; Tian, Y. S.; Liu, B.; Sun, J.; Smilgies, D. M.; Thoroddsen, S.; Amassian, A.; Zhao, K.; Liu, S. Z., Phase Transition Control for High-Performance Blade-Coated Perovskite Solar Cells. *Joule* **2018**, 2 (7), 1313-1330. [Note: Simultaneous solvent evaporation and crystallization with precursor deposited at 125 °C.]

18. Zhong, Y. F.; Munir, R.; Li, J. B.; Tang, M. C.; Niazi, M. R.; Smilgies, D. M.; Zhao, K.; Arnassian, A., Blade-Coated Hybrid Perovskite Solar Cells with Efficiency > 17%: An In Situ Investigation. *Acs Energy Lett* **2018**, 3 (5), 1078-1085. [Note: Simultaneous solvent evaporation and crystallization with precursor deposited at 130 °C.]

19. Yin, J.; Lin, Y. C.; Zhang, C. Q.; Li, J.; Zheng, N. F., Growth-Dynamic-Controllable Rapid Crystallization Boosts the Perovskite Photovoltaics' Robust Preparation: From Blade Coating to Painting. *Acs Appl Mater Inter* **2018**, 10 (27), 23103-23111. [Note: Simultaneous solvent evaporation and crystallization with precursor deposited at 145 °C.]

20. Kong, W. G.; Wang, G. L.; Zheng, J. M.; Hu, H.; Chen, H.; Li, Y. L.; Hu, M. M.; Zhou, X. Y.; Liu, C.; Chandrashekar, B. N.; Amini, A.; Wang, J. B.; Xu, B. M.; Cheng, C., Fabricating High-Efficient Blade-Coated Perovskite Solar Cells under Ambient Condition Using Lead Acetate Trihydrate. *Sol Rrl* **2018**, 2 (3). [Note: Simultaneous solvent evaporation and crystallization with precursor deposited at 135 °C.]

21. Heo, Y. J.; Kim, J. E.; Weerasinghe, H.; Angmo, D.; Qin, T. S.; Sears, K.; Hwang, K.; Jung, Y. S.; Subbiah, J.; Jones, D. J.; Gao, M.; Kim, D. Y.; Vak, D., Printing-friendly sequential deposition via intra-additive approach for roll-to-roll process of perovskite solar cells. *Nano Energy* **2017**, 41, 443-451. [Note: N_2-blowing assisted crystallization with precursor deposited at 140 °C.]

22. Mallajosyula, A. T.; Fernando, K.; Bhatt, S.; Singh, A.; Alphenaar, B. W.; Blancon, J. C.; Nie, W.; Gupta, G.; Mohite, A. D., Large-area hysteresis-free perovskite solar cells via temperature controlled doctor blading under ambient environment. *Appl Mater Today* **2016**, 3, 96-102. Simultaneous solvent evaporation and crystallization with precursor deposited at 150 °C.]
